# Association between Gut Microbiota and Body Composition in Japanese General Population: A Focus on Gut Microbiota and Skeletal Muscle

**DOI:** 10.3390/ijerph19127464

**Published:** 2022-06-17

**Authors:** Yoshikuni Sugimura, Akira Kanda, Kaori Sawada, Kyi Mar Wai, Asano Tanabu, Naoki Ozato, Tatsuyuki Midorikawa, Takayoshi Hisada, Shigeyuki Nakaji, Kazushige Ihara

**Affiliations:** 1 Department of Innovation Center for Health Promotion, Graduate School of Medicine, Hirosaki University 5 Zaifu-cho, Hirosaki 036-8562, Japan; y.sugimura@hirosaki-u.ac.jp (Y.S.); nakaji@hirosaki-u.ac.jp (S.N.); 2 Department of Social Medicine, Graduate School of Medicine, Hirosaki University 5 Zaifu-cho, Hirosaki 036-8562, Japan; iwane@hirosaki-u.ac.jp (K.S.); kyimar@hirosaki-u.ac.jp (K.M.W.); mimiruru33@gmail.com (A.T.); tmidori@lion.co.jp (T.M.); 3 Department of Nutrition, Faculty of Health Sciences, Aomori University of Health and Welfare, 58-1 Mase, Hamadate, Aomori 030-8505, Japan; a_kanda@auhw.ac.jp; 4 Health & Wellness Products Research Laboratories, Kao Corporation, 2-1-3 Bunka, Sumida-ku, Tokyo 131-8501, Japan; oozato.naoki@kao.com; 5 Research and Development Headquarters, Lion Corporation, Odawara 256-0811, Japan; 6 TechnoSuruga Laboratory Co., Ltd., 388-1 Nagasaki, Shimizu-ku, Shizuoka 424-0065, Japan; th_1005@tecsrg.co.jp

**Keywords:** sarcopenia, gut microbiota, skeletal muscle mass, skeletal muscle mass index

## Abstract

This study aimed to investigate the gut microbial genera associated with skeletal muscle mass, using a large-scale survey from the standpoint of preventing sarcopenia. A total of 848 participants were included in the analysis. The mean (SD) ages of men (*n* = 353) and women (*n* = 495) were 50.0 (12.9) years and 50.8 (12.8) years, respectively. Body composition was assessed using appendicular skeletal muscle mass/body weight (ASM/BW), ASM, and BW. Additionally, the relationship between gut microbial genera and body composition was analyzed. The means (SD) of ASM/BW were 34.9 (2.4) % in men and 29.4 (2.9) % in women. *Blautia* and *Bifidobacterium* were positively associated with ASM/BW only in men (*Blautia*: β = 0.0003, *Bifidobacterium*: β = 0.0001). However, *Blautia* was negatively associated with BW (β = −0.0017). *Eisenbergiella* was positively associated with ASM/BW (β = 0.0209) and negatively associated with BW (β = −0.0769) only in women. Our results indicate that *Blautia*, *Bifidobacterium* and *Eisenbergiella*, which are positively associated with ASM/BW, might help increase skeletal muscle mass. ASM/BW may clarify the relationship between gut microbiota and skeletal muscle mass without being affected by obesity or excess body fat mass.

## 1. Introduction

Sarcopenia is a symptomatic condition characterized by decreased muscle strength and physical function due to excessive loss of skeletal muscle mass with aging. Sarcopenia is accompanied by poor physical balance, gait disturbances, cane use, and falls [1]. Skeletal muscle mass has been reported to decrease by 5–10% of its youthful level by the age of 50 and decrease by 30–40% between the age of 50–80 years [2]. Functional impairment (inability to stand up from a chair or lift an object) may occur when skeletal muscle mass is reduced by 20–30% of its youthful level [3]. Moreover, such a decrease in skeletal muscle mass is associated with an increase in future all-cause mortality; therefore, a decrease in skeletal muscle must be prevented [4].

Recent studies have reported that gut microbiota is associated with skeletal muscle mass in animals [5]. Siddharth et al. reported that the amount of gastrocnemius muscle was reduced to a greater extent in aged rats with sarcopenia than in normal adult rats. This reduction occurs with changes in the composition of gut microbiota [6]. Munukka et al. reported that inoculation with *Faecalibacterium prausnitzii* increases muscle mass in mice [7]. Lahiri et al. reported that transplantation of stool from mice with gut microbiota into germ-free mice increased skeletal muscle mass [8].

Some studies in humans have also suggested an association between gut microbiota and skeletal muscle mass [9,10,11]. Among the residents of welfare facilities for older adults, those who were frailer had a lower relative abundance of the major gut microbiota [9]. The sarcopenia group and possible sarcopenia group had a lower relative abundance of *Roseburia* and *Eubacterium* compared to the healthy control group [10]. Furthermore, it was reported that when frail older adults took *LactbacillusTWK10*, the muscle mass increased without increasing body fat percentage [12]. One study in younger adults reported that male athletes had a higher diversity of gut microbiota than male non-athletes, with a higher relative abundance of *Akkermansia muciniphila* [13]. Another study of women aged 18 to 40 years with normal BMI reported that the relative abundance of *Coprococcus* was positively correlated with the skeletal muscle index [11].

Previous studies examining a small number of frail older adults [9,10] and younger adults (18–40 years old) [11] were conducted for the establishment of microbial genera that affect skeletal muscle mass. Therefore, a large-scale study of the general population considering individuals’ various health conditions is needed to overcome the previous limitation of a smaller population.

Each skeletal muscle mass index has its own special characteristics; therefore, it was selected according to the purpose of the research and evaluation. Some studies of skeletal muscle in relation to gut microbiota used the appendicular skeletal muscle mass index (ASMI), calculated as the appendicular skeletal muscle mass (ASM) divided by height squared (ASM/height^2^) (kg/m^2^) [10,14]. However, ASMI is highly correlated with body weight (BW). Therefore, body fat should be considered in overweight and obese individuals [15]. In addition, the height of adults gradually shortens with age. Therefore, ASMI values increase, even if BW and muscle mass remain constant. The appendicular skeletal muscle mass divided by BW (ASM/BW) is a marker considered for studying the practical definition of sarcopenia. Sarcopenia categorized by ASM/BW had a worse physiological status than that categorized by ASMI [16]. In addition, a previous report showed that ASM/BW was negatively associated with insulin resistance, whereas ASMI was positively associated with insulin resistance [17]. These studies suggest that ASM/BW indicates the relationship between skeletal muscle mass and health status better than ASMI [16,17]. In addition, the values of ASM/BW were not affected by body fat or change in height. Thus, ASM/BW may be a more accurate marker for the assessment of skeletal muscle mass than ASMI in studies involving participants with a wider age range.

More comprehensive knowledge of the gut microbiota that lead to an increase in skeletal muscle mass will contribute to the prevention of sarcopenia. However, to our knowledge, no previous studies have used ASM/BW to investigate the relationship be-tween gut microbiota and skeletal muscle mass. Although there are several methods to measure ASM, such as magnetic resonance, computed tomography, dual-energy X-ray absorptiometry (DXA), and bioelectrical impedance analysis (BIA), we adopted the BIA method to assess ASM in this study [18]. BIA is a non-invasive, relatively less costly, and technologically friendly method with high reliability and validity. It is widely used in clinical and practical settings, and is suitable for large-scale surveys [19]. We conducted a large-scale survey of local residents of a wide range of ages to establish the gut microbial genera associated with skeletal muscle mass using ASW/BW as an index of sarcopenia by a non-invasive measurement.

## 2. Materials and Methods

### 2.1. Study Participants

**Figure 1:**
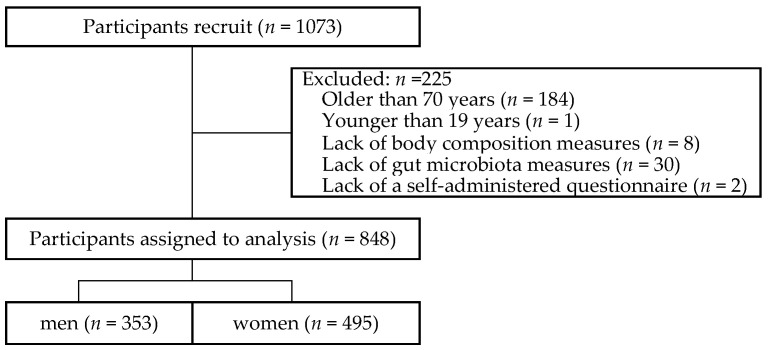
Participant flow diagram.

This cross-sectional study focused on the relationship between gut microbiota and skeletal muscle mass in 1073 healthy residents aged between 19 and 93 years who were living in the Iwaki area of Hirosaki City, Aomori Prefecture, and participated in the 2017 Iwaki Health Promotion Project [20]. One hundred eighty-four participants aged 70 years or older were excluded from the analysis because healthy gut microbiota was affected by age-related physiological and behavioral changes after age 70 [21]. Participants in the following categories were also excluded: (i) younger than 19 years (*n* = 1), (ii) lack of body composition measures (*n* = 8), (iii) lack of gut microbiota measures (*n* = 30), and (iv) lack of a self-administered questionnaire (*n* = 2). Finally, a total of 848 participants were included in the analysis (Figure 1).

### 2.2. Measurements of Body Composition

Body composition in this study was measured by BIA using MC-190 (Tanita, Tokyo, Japan) in a standing position. The BIA is a multi-frequency, 8-electrode system that can provide estimates of muscle mass and body fat content for the entire body, trunk, and extremities [22]. Participants were asked to stand barefoot on toe-and-heel electrodes, and to hold the handgrips with arms hanging down a few centimeters from the body. Multiple frequencies (5, 50, 250, 500 kHz, 90 µA or less) were supplied, and values of resistance (75–1500 Ω) and reactance were measured between hands and feet in the device [23]. A prediction model setting height, weight, age, body type information (standard/athlete), value of reactance extracted from BIA, and the outcome from BIA using multiple frequencies as explanatory variables was implemented to estimate the muscle mass that could be measured using DXA and dilution methods [23]. The values of muscle mass were highly correlated with the measurements by DXA (total muscle mass: r = 0.96) [23]. Previous studies reported that the intraclass correlation of total fat-free mass (kg) was 0.95 [24]. However, the values are somewhat affected by changes in body water percentage and body temperature during the day. Values are also affected by activities such as eating, sleeping, sweating, urinating, and drinking alcohol [23]. Therefore, participants were asked to skip breakfast on the day of body composition measurements to minimize diurnal behavioral variation in values obtained by BIA. To adjust the condition of body water, body composition measurements were obtained soon after urine sampling. The BIA readings differ by different BIA instruments, as the instruments are dependent on different technologies [18,25,26,27] and predictive equations [28]. To minimize the variabilities by measuring instrument, the same instrument was used throughout the study.

Body composition was assessed based on ASM/BW, ASM, and BW. ASM/BW was used as the skeletal muscle index [3,16]. ASM (kg) and ASM/BW (%) were calculated as follows: ASM (kg) = right arm muscle (kg) + left arm muscle (kg) + right leg muscle (kg) + left leg muscle (kg); ASM/BW (%) = ASM (kg)/BW (kg) × 100.

### 2.3. Measurements of the Gut Microbiota

Fecal sampling kits were distributed to the participants before the health examination. Sampling kits contained guanidine thiocyanate solution (100 mM Tris-HCl (pH 8.0), 40 mM Tris-EDTA (pH 8.0), 4 M guanidine thiocyanate, and 0.001% bromothymol blue) for the stability of the gut microbiota composition (Techno Suruga Laboratory Co., Ltd., Shizuoka, Japan). Fecal samples were collected at home by the participants using the kit within 3 days prior to the health examination and handed in on the day of the health examination. Participants were instructed to defecate on toilet paper, scoop the stool with the provided spoon, and return it to the container. They were instructed to keep the samples in their home refrigerators until the day of the health examination. Fecal samples were stored at 4 °C until DNA extraction for 3 months at Techno Suruga Laboratory [29,30,31]. DNA of the gut microbiota in the fecal cell suspension was extracted using the zirconium bead disruption method. For DNA purification, an automated nucleic acid extraction system (Precision System Science, Chiba, Japan) and MagDEA DNA200 (Precision System Science) were used. For DNA amplification, the concentration of the purified DNA was adjusted to 10 ng/μL using the NanoDrop absorption method.

The V3-V4 region of the prokaryotic 16S rRNA in the gut microbiota was amplified using universal primer sets [32]. Amplified DNA was sequenced using the Illumina MiSeq sequencing system and MiSeq Reagent Kit v3 (Illumina, San Diego, CA, USA).

The partial base sequence of 16SrDNA (approximately 380–430 bp) clustered at a homology rate of more than 97% using VSEARCH (version 2.4.3). The clusters identified with a confidence value of less than 0.8 were grouped as an unclassified taxon. The taxa of clusters were identified using the standard classification predicted by the RDP classifier (commit hash: 701e229dde7cbe53d4261301e23459d91615999d). The numbers of each taxon were calculated as read counts of the partial base sequence of 16SrDNA. Regarding intra-individual variations, no time trend was observed in response to both seasonal and diurnal alterations [33].

### 2.4. Self-Reported Questionnaire

Daily intakes of protein, fat, carbohydrates, alcohol, and total dietary fiber were calculated from the results of the brief-type self-administered diet history questionnaire (BDHQ) [34]. Smoking habits (c/d), physical activity (min/week), sleep time (min/day), and habitual medicine use (yes/no) were surveyed using self-administered questionnaires.

### 2.5. Statistical Analysis

All data analyses were conducted according to sex. The characteristics of the participants are shown as mean ± standard deviation for numerical variables and percentage for nominal variables. Analyses of gut microbiota were performed at the phylum and genus levels of read counts. Subsequent analyses were conducted for genera whose mean read counts were 1.0 or higher. Finally, 90 of the 319 identified genera were included in the analysis. Univariate associations of the read counts of gut microbial genera with ASM/BW, ASM, and BW were analyzed using Spearman’s correlation. In addition, false discovery rate (FDR) correction was used to determine the gut microbial genera associated with body composition. FDR was controlled by the Benjamini–Hochberg procedure in order to correct for multiple testing, and was considered significant at <0.05 [35]. The relationship between the read counts of gut microbial genera (outcome) and ASM/BW, ASM, and BW (exposure) was investigated by multivariate analysis using a linear regression model (stepwise method). This multivariate analysis was adjusted for age, nutrient intake (protein, fat, carbohydrate, total dietary fiber, and alcohol), habitual medicine use, smoking habits, physical activity, and sleep duration. Statistical analyses were performed using SPSS (version 25; SPSS Inc., Chicago, IL, USA). Statistical significance was set at *p* < 0.05.

## 3. Results

### 3.1. Characteristics of participants

The characteristics of the study participants are presented in Table 1. The mean age of men (*n* = 353) was 50.0 ± 12.9 years, and the mean age of women (*n* = 495) was 50.8 ± 12.8 years. Approximately half of both men and women were taking medications for lifestyle-related diseases.

We examined the correlation between body composition variables. BW was negatively correlated with ASM/BW (men: r = −0.372, women: r = −0.683). BW showed a strong positive correlation with ASM (men: r = 0.861, women: r = 0.750). ASM and ASM/BW showed a weak positive correlation (r = 0.142) in men but not in women.

### 3.2. Gut Microbiota Composition of the Participants

Table 2 shows the mean read counts of the phyla and genera of the gut microbiota, which were significantly correlated with body composition. The mean read count of Firmicutes was the highest (approximately 10,700), followed by Bacteroidetes (approximately 6000), Actinobacteria (approximately 2200), and Proteobacteria (approximately 550). The mean read count of Firmicutes was significantly higher in women than in men (*p* = 0.014), whereas Bacteroidetes (*p* = 0.056) and Proteobacteria (*p* < 0.001) were lower in women than in men.

At the genus level, the mean read count of *Bacteroides* was the highest (approximately 3800), followed by *Blautia* (approximately 1600) and *Bifidobacterium* (approximately 1550). The mean read count of *Bacteroides* was significantly higher in women than in men (*p* = 0.014), whereas those of *Blautia* and *Bifidobacterium* did not significantly differ between men and women (*Blautia*: *p* = 0.063, *Bifidobacterium*: *p* = 0.189).

### 3.3. Association between Gut Microbiota and Body Composition

**Figure 2:**
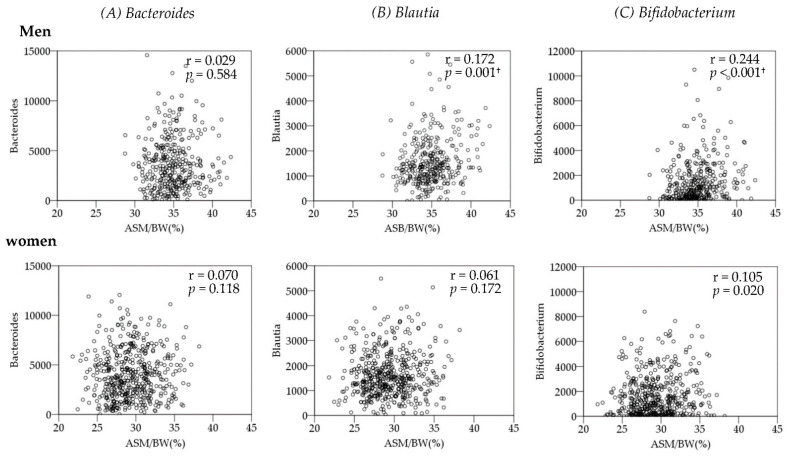
Spearman’s correlation between mean read count and ASM/BW of the three genera with the highest read counts. † *p*-values that passed the FDR 0.05 threshold.

The correlation coefficients between ASM/BW and the genera with the three highest mean read counts are shown in Figure 2. ASM/BW was positively correlated with *Blautia* and *Bifidobacterium* in men (*Blautia*; r = 0.172, *p* = 0.001, *Bifidobacterium*; r = 0.244, *p* < 0.001).

Details of other correlations of gut microbiota with body composition are shown in Table 3. The significant correlation of two genera in men, and five genera in women, with ASM/BW, remained after FDR was corrected to <0.05. The highest positive correlation of ASM/BW was shown with *Bifidobacterium* (r = 0.244, *p* < 0.001) in men and with *Eisenbergiella* (r = 0.183, *p* < 0.001) in women. The highest negative correlation of ASM/BW was observed with *Dorea* (r = −0.136, *p* < 0.002) in women. In addition, the highest positive correlation of BW was observed with *Dorea* (r = 0.158, *p* < 0.001).

Multiple linear regression analyses (stepwise method) were performed, with each body composition as a dependent variable. Each of the seven genera was entered as an independent variable, with possible confounders. Significant results for the genera are shown in Table 4. *Blautia* (β = 0.0003, *p* = 0.010) and *Bifidobacterium* (β = 0.0001, *p* = 0.038) in men were associated with ASM/BW. *Dorea* (β = −0.0016, *p* = 0.014) and *Eisenbergiella* (β = 0.0209, *p* = 0.038) in women were associated with ASM/BW. *Blautia* in men (β = −0.0017, *p* = 0.006), and *Dorea* (β = 0.0056, *p* = 0.016) and *Eisenbergiella* (β = −0.0769, *p* = 0.034) in women, were associated with BW.

## 4. Discussion

The aim of this study was to clarify the gut microbiota associated with skeletal muscle mass and skeletal muscle index, in relation to the prevention of sarcopenia. Our study revealed that the read counts of *Blautia*, *Bifidobacterium*, *Dorea* and *Eisenbergiella* were associated with ASM/BW in a large-scale survey. In particular, the increased read counts of *Blautia*, *Bifidobacterium*, and *Eisenbergiella* appeared to be associated with a larger skeletal muscle mass in men. These gut microbial genera were different from those reported in previous studies, in which skeletal muscle was assessed with ASMI.

The higher read count of *Blautia* in men was accompanied by a higher ASM/BW. *Blautia*, a dominant bacterium in the intestine, produces acetic acid. Maruta et al. reported that acetic acid activates G-protein receptors and increases production of muscle-related proteins using myoblasts [36]. In human studies, an association of *Blautia* with skeletal muscle has not been reported. However, the association of *Blautia* with inflammatory and metabolic disorders has been reported [37]. The relative abundance of *Blautia* was lower in patients with type 2 diabetes and was negatively correlated with HbA1c [38]. An increase in the relative abundance of *Blautia* was positively correlated with the maintenance of glucose and lipid homeostasis [39]. However, loss of skeletal muscle mass, such as sarcopenia, was reported to lower efficiency of glucose uptake from the blood, leading to type 2 diabetes [40]. Therefore, the higher read count of *Blautia* was possibly associated with an increased skeletal muscle mass, leading to the prevention of sarcopenia and inflammatory and metabolic diseases.

A higher read count of *Bifidobacterium* was associated with a higher ASM/BW in men in the multivariate analysis. In mice transplanted with *Bifidobacterium longum BL986*, the muscle mass/body weight ratio and grip strength increased without weight gain, suggesting that *Bifidobacterium* can regulate muscle mass in mice [41]. Obese human participants who were fed *Bifidobacterium breve B-3* showed a decrease in body fat and an increase in muscle mass without a significant change in BMI [42]. These results support the significant association between *Bifidobacterium* and ASM/BW in men in our study. The association, however, was not shown in women; therefore, further studies on sex differences of such functional mechanisms are needed in the future.

A higher read count of *Eisenbergiella* was associated with a higher ASM/BW in women, as well as with a lower BW in women. *Eisenbergiella* is a bacterial genus that could be related to the development of obesity and other pro-inflammatory diseases in women [43]. In addition, the relative abundance of *Eisenbergiella* in male bodybuilders was higher than in distance runners and healthy sedentary men [44]. Our results suggest that *Eisenbergiella* is associated with skeletal muscle mass, strengthening previous findings on this topic.

A higher read count of *Dorea* in women was accompanied by lower ASM/BW. The association of *Dorea* with skeletal muscle has not yet been studied, but the decrease in the relative abundance of *Dorea* has prevented cardiac risk factors, such as higher BMI, abdominal circumference, blood pressure, and triglycerides [45]. A higher relative abundance of *Dorea* was associated with higher fasting blood glucose levels [46]. Therefore, *Dorea* may be associated with metabolic diseases. Our results, which strengthen previous findings, suggest that *Dorea* is associated with skeletal muscle mass.

The gut microbial genera and composition associated with ASM/BW differed between men and women [47,48], and sex hormones are considered major determinants of the composition of the gut microbiota [49]. Sex hormones also contribute to differences in muscle synthesis and fat distribution between men and women [50]. The sex difference in the association between gut microbial genera and ASM/BW might be explained by adjusting for confounders, such as sex hormones.

This study has two advantages. First, our large-scale survey of community dwellers enabled us to examine the association of gut microbiota with ASM/BW stratified by sex. Second, we used ASM/BW, which can indicate healthy skeletal muscle mass. To the best of our knowledge, previous studies on gut microbiota and skeletal muscle mass were conducted in less than 100 individuals, irrespective of sex. In addition, participants in previous studies were over 70 years of age in institutions or professional rugby athletes.

Our study has some limitations. First, this study was cross-sectional and could not evaluate cause and effect relationships. Second, the gut microbiota may affect skeletal muscle mass and muscle function through inflammation, immunity, energy metabolism, and insulin sensitivity. We are ready to investigate the effect of inflammatory substances on gut microbiota by blood test. Third, our study was conducted in one community, and results from other communities or regions are necessary. Fourth, comparisons among different age groups with large numbers of participants are necessary because gut microbiota and skeletal muscle mass change with age. Fifth, nutrient intake was estimated using a self-administered food frequency questionnaire. Chemical analyses of nutrients in meals are recommended to obtain more accurate results. The use of portable electric devices to measure nutritional intake and physical activity will help obtain further results. Sixth, our study could not be analyzed at the strain or species level. Bacterial strains and species are diverse within the genus, and analyses at and below the species level may identify more specific species or strains relating to skeletal muscle.

## 5. Conclusions

Our study with participants of a large-scale health check-up revealed that *Blautia* and *Bifidobacterium* were positively correlated with ASM/BW only in men. In addition, *Eisenbergiella* was positively correlated with ASM/BW only in women. The relationship of ASM/BW with *Blautia*, *Bifidobacterium* and *Eisenbergiella* has not been shown in previous studies. These gut microbial genera may help increase skeletal muscle mass. ASM/BW may clarify the relationship between gut microbiota and skeletal muscle mass without being affected by overweight or body fat mass, even in individuals with obesity or excess body fat mass.

## Figures and Tables

**Table 1:** Characteristics of the study participants.

	Men	Women
	*n* = 353	*n* = 495
	Mean ± SD	Mean ± SD
Age (years)	50.0 ± 12.9	50.8 ± 12.8
**Body composition**		
Body weight (kg)	69.4 ± 11.3	54.7 ± 9.4
ASM (kg)	24.1 ± 3.5	15.9 ± 1.9
ASM/BW (%)	34.9 ± 2.4	29.4 ± 2.9
**Nutrition**		
Protein intake (g/d)	75.8 ± 25.8	64.4 ± 21.7
Fat intake (g/d)	56.3 ± 19.5	50.6 ± 16.7
Carbohydrate intake (g/d)	290.4 ± 85.9	217.4 ± 64.4
Total dietary fiber intake (g/d)	11.3 ± 4.6	10.3 ± 3.9
Alcohol intake (g/d)	22.6 ± 26.4	5.4 ± 12.2
**Lifestyle**		
Smoking (s/d)	7.4 ± 13.6	2.3 ± 7.9
Physical activity (min/wk)	52.8 ± 149.2	35.7 ± 103.6
Sleep time (min/d)	419.1 ± 64.3	400.6 ± 64.5
Habitual medicine use (No, %)	194 (55.0)	245 (49.5)
(Yes, %)	159 (45.0)	250 (50.5)

All values are expressed as mean ± standard deviation unless indicated otherwise. There were 20 deficiencies in physical activity. Abbreviations: SD: standard deviation, ASM = appendicular skeletal muscle mass, ASM/BW (%) = appendicular skeletal muscle mass/body weight × 100, g/d: grams per day, s/d: sticks per day, min/wk: minutes per week, min/d: minutes per day.

**Table 2:** Gut microbiota composition.

	Men (*n* = 353)	Women (*n* = 495)	*p*-Value
	Mean ± SD	Mean ± SD	
**Phylum**			
Firmicutes	10442.9 ± 3334.0	10986.7 ± 3103.6	0.014
Bacteroidetes	6324.0 ± 3264.8	5802.2 ± 2645.7	0.056
Actinobacteria	2214.3 ± 1944.5	2232.3 ± 1813.7	0.782
Proteobacteria	632.1 ± 873.4	460.7 ± 560.7	<0.001
**Genera**			
B_*Bacteroides*	3663.8 ± 2595.0	3993.9 ± 2442.8	0.014
F_*Blautia*	1550.8 ± 937.9	1658.4 ± 1001.2	0.063
A*_Bifidobacterium*	1498.5 ± 1685.8	1619.7 ± 1609.3	0.189
F_*Fusicatenibacter*	521.1 ± 510.5	536.1 ± 556.4	0.707
B*_Parabacteroides*	420.2 ± 597.9	437.1 ± 509.9	0.019
F_*Gemmiger*	345.3 ± 400.9	461.1 ± 443.4	<0.001
F_*Ruminococcus2*	343.2 ± 391.6	341.0 ± 384.8	0.500
F_*Dorea*	203.0 ± 184.7	177.1 ± 180.1	0.009
F_*Clostridium XlVa*	145.6 ± 140.5	170.7 ± 153.0	0.002
F*_Clostridium IV*	100.8 ± 184.9	178.5 ± 309.5	<0.001
F_*Holdemanella*	147.0 ± 306.3	120.3 ± 306.2	0.081
F_*Clostridium XVIII*	122.1 ± 204.0	130.8 ± 186.8	0.071
F_*Coprococcus*	119.2 ± 155.7	124.3 ± 176.3	0.429
P_*Escherichia/Shigella*	91.5 ± 408.7	110.1 ± 358.2	0.752
F*_Dialister*	82.1 ± 165.8	106.0 ± 193.3	0.001
F_*Lactobacillus*	114.0 ± 540.1	40.5 ± 142.9	0.855
P*_Parasutterella*	66.5 ± 135.9	77.1 ± 151.2	0.708
B_*Paraprevotella*	63.2 ± 145.0	45.8 ± 125.5	0.002
F_*Bacillus*	38.1 ± 119.7	41.1 ± 95.9	0.071
F*_Clostridium XlVb*	40.7 ± 61.3	39.1 ± 72.8	0.964
B*_Acidaminococcus*	42.6 ± 107.5	29.7 ± 101.9	0.001
F_*Flavonifractor*	29.6 ± 39.5	41.6 ± 42.9	<0.001
B_*Barnesiella*	23.1 ± 62.1	35.9 ± 78.7	0.338
A_*Eggerthella*	23.0 ± 43.2	36.6 ± 54.1	<0.001
F_*Odoribacter*	21.9 ± 47.6	30.1 ± 42.4	<0.001
F_*Erysipelotrichaceae_incertae_sedis*	20.1 ± 46.2	28.5 ± 84.7	0.195
P*_Bilophila*	21.9 ± 37.9	27.2 ± 37.3	0.109
P*_Enterobacter*	21.0 ± 146.6	5.8 ± 56.8	0.291
F_*Terrisporobacter*	14.6 ± 57.3	14.0 ± 57.6	0.290
B_*Butyricimonas*	12.1 ± 30.0	13.7 ± 26.3	0.158
A_*Senegalimassilia*	12.7 ± 39.0	8.9 ± 30.4	<0.001
B_*Allisonella*	9.3 ± 17.5	5.3 ± 12.5	<0.001
P_*Raoultella*	6.7 ± 39.0	6.9 ± 67.9	0.800
F_*Intestinimonas*	5.1 ± 13.8	8.6 ± 16.8	<0.001
P_*Succinivibrio*	8.2 ± 88.8	1.5 ± 23.0	0.219
P_*Desulfovibrio*	4.4 ± 21.5	2.2 ± 12.6	0.389
A_*Olsenella*	3.0 ± 12.0	1.9 ± 8.4	0.003
A_*Gordonibacter*	1.6 ± 3.6	3.0 ± 4.9	<0.001
A_*Rothia*	2.4 ± 5.6	1.9 ± 3.7	0.131
F_*Eisenbergiella*	1.0 ± 4.1	3.3 ± 11.6	<0.001
P*_Serratia*	0.0 ± 0.3	2.3 ± 48.2	0.410
F_*Pseudoflavonifractor*	1.1 ± 8.7	1.0 ± 2.8	0.001

Only the human gut microbiota genera significantly associated with body composition in men and women are shown. Mean ± standard deviations are presented for continuous variables. *p*-values are presented for the differences between men and women. *p* < 0.05, Mann–Whitney U-test.

**Table 3:** Spearman’s correlation between gut microbiota and body composition.

	Men (*n* = 353)						Women (*n* = 353)					
	ASM/BW (%)		ASM (kg)		Body Weight (kg)		ASM/BW (%)		ASM (kg)		Body Weight (kg)	
	r	*p*	r	*p*	r	*p*	r	*p*	r	*p*	r	*p*
B*_Bacteroides*	0.029	0.584	0.086	0.105	0.079	0.140	0.070	0.118	−0.065	0.147	−0.094	0.037
F*_Blautia*	**0.172**	**0.001 ^†^**	−0.014	0.800	−0.100	0.061	0.061	0.172	−0.082	0.068	−0.094	0.036
A_*Bifidobacterium*	**0.244**	**0.000 ^†^**	0.115	0.031	−0.017	0.745	0.105	0.020	0.125	0.005	0.012	0.792
F*_Fusicatenibacter*	0.007	0.898	0.109	0.041	0.074	0.165	−0.09	0.045	0.030	0.503	0.094	0.036
B_*Parabacteroides*	0.108	0.043	0.005	0.933	−0.029	0.583	0.108	0.016	0.096	0.033	−0.003	0.939
F_*Gemmiger*	0.113	0.033	0.097	0.069	0.023	0.667	0.066	0.144	0.011	0.811	−0.035	0.442
F*_Ruminococcus2*	−0.082	0.122	0.117	0.028	0.137	0.010	−0.068	0.129	0.09	0.046	0.121	0.007
F_*Dorea*	−0.011	0.830	0.103	0.053	0.102	0.056	**−0.136**	**0.002 ^†^**	0.086	0.054	**0.158**	**0.000 ^†^**
F*_Clostridium XlVa*	−0.039	0.461	0.046	0.387	0.062	0.248	0.066	0.145	−0.074	0.101	−0.106	0.018
F_*Clostridium IV*	0.032	0.544	0.018	0.738	−0.002	0.965	0.042	0.349	0.122	0.007	0.076	0.092
F_*Holdemanella*	−0.099	0.062	0.045	0.404	0.104	0.051	−0.111	0.013	0.021	0.634	0.077	0.087
F*_Clostridium XVIII*	0.066	0.219	−0.040	0.455	−0.061	0.251	−0.028	0.530	−0.108	0.016	−0.051	0.254
F*_Coprococcus*	−0.044	0.413	0.083	0.120	0.113	0.033	−0.118	0.009	0.028	0.527	0.093	0.039
P_*Escherichia/Shigella*	−0.048	0.364	0.005	0.925	0.031	0.568	0.122	0.007	0.022	0.622	−0.061	0.173
F*_Dialister*	0.032	0.553	0.050	0.345	0.036	0.505	−0.107	0.017	0.083	0.066	0.126	0.005
F*_Lactobacillus*	−0.14	0.008	−0.035	0.508	0.050	0.353	−0.076	0.093	0.070	0.118	0.099	0.027
P*_Parasutterella*	0.024	0.658	0.115	0.031	0.088	0.097	0.014	0.760	0.068	0.131	0.041	0.362
B_*Paraprevotella*	0.013	0.812	−0.002	0.972	0.007	0.901	−0.099	0.027	0.049	0.273	0.094	0.036
F_*Bacillus*	−0.034	0.521	−0.053	0.319	−0.021	0.695	−0.043	0.342	−0.102	0.024	−0.030	0.512
F_*Clostridium XlVb*	−0.121	0.023	0.043	0.420	0.091	0.089	0.014	0.758	0.063	0.164	0.046	0.308
F_*Acidaminococcus*	−0.017	0.757	0.096	0.071	0.137	0.010	−0.088	0.050	0.024	0.599	0.073	0.103
F*_Flavonifractor*	0.138	0.009	0.061	0.255	−0.007	0.891	**0.157**	**0.000 ^†^**	0.004	0.930	−0.089	0.047
B_*Barnesiella*	−0.033	0.538	0.032	0.543	0.041	0.442	0.004	0.936	0.096	0.032	0.080	0.077
A_*Eggerthella*	0.134	0.011	−0.010	0.851	−0.087	0.102	**0.143**	**0.001 ^†^**	−0.012	0.793	−0.093	0.038
B_*Odoribacter*	0.014	0.786	0.078	0.142	0.060	0.259	0.086	0.055	0.136	0.003	0.052	0.245
F_*Erysipelotrichaceae_incertae_sedis*	0.110	0.038	0.014	0.792	−0.030	0.572	**0.146**	**0.001 ^†^**	−0.006	0.902	−0.104	0.020
P*_Bilophila*	0.014	0.787	0.058	0.280	0.078	0.144	0.056	0.211	0.115	0.010	0.059	0.192
F*_Eisenbergiella*	0.049	0.363	0.019	0.717	−0.004	0.935	**0.183**	**0.000 ^†^**	−0.025	0.585	−0.135	0.003

The correlated gut microbiota and body composition of men and women are shown. r: Spearman’s correlation coefficient. † *p* values that passed the FDR 0.05 threshold. Abbreviations: ASM (kg) = appendicular skeletal muscle mass, ASM/BW (%) = appendicular skeletal muscle mass/body weight × 100.

**Table 4:** Association between gut microbiota and body composition.

	Explanatory Variables	ASM/BW (%)	ASM (kg)	Body Weight (kg)
		(95%Cl)	(95%Cl)	(95%Cl)
**Men**	F*_Blautia*	0.0003 (0.0001, 0.0006) *		−0.0017 (−0.0029, −0.0005) **
	age	−0.08 (−0.1, −0.06) ***		−0.16 (−0.25, −0.07) **
	sleep time			−0.03 (−0.05, −0.01) **
	fat intake			0.09 (0.03, 0.15) **
	A_*Bifidobacterium*	0.0001 (0.00001, 0.0003) *		
	age	−0.08 (−0.1, −0.06) ***		
**Women**	F*_Dorea*	−0.0016 (−0.0029, −0.0003) *		0.0056 (0.001, 0.0102) *
	age	−0.11 (−0.13, −0.09) ***		
	F_*Flavonifractor*		−0.0056 (−0.0094, −0.0018) **	−0.0271 (−0.0463, −0.0079) **
	age		−0.06 (−0.08, −0.05) ***	
	A*_Eggerthella*		−0.0044 (−0.0073, −0.0015) **	−0.0207 (−0.0358, −0.0055) **
	age		−0.062 (−0.07, −0.05) ***	
	F_*Erysipelotrichaceae_incertae_sedis*		−0.0022 (−0.0041, −0.0004) *	
	age		−0.06 (−0.07, −0.05) ***	
	F*_Eisenbergiella*	0.0209 (0.0011, 0.0407) *		−0.0769 (−0.1481, −0.0057) *
	age	−0.113 (−0.132, −0.094) ***		
	total fiber	0.063 (0.002, 0.125) *		

Abbreviations: F, Firmicutes; A, Actinobacteria; β, standardized regression; Cl, Confidence interval. * *p* < 0.05; ** *p* < 0.01; *** *p* < 0.001.

## Data Availability

The data presented in this study are available upon request from the corresponding author. The data are not publicly available due to the test sample patent and the participants’ privacy and confidentiality.
